# The interaction of heme with plakortin and a synthetic endoperoxide analogue: new insights into the heme-activated antimalarial mechanism

**DOI:** 10.1038/srep45485

**Published:** 2017-04-06

**Authors:** Marco Persico, Roberto Fattorusso, Orazio Taglialatela-Scafati, Giuseppina Chianese, Ivan de Paola, Laura Zaccaro, Francesca Rondinelli, Marco Lombardo, Arianna Quintavalla, Claudio Trombini, Ernesto Fattorusso, Caterina Fattorusso, Biancamaria Farina

**Affiliations:** 1 University of Naples “Federico II”, Department of Pharmacy, Via D. Montesano 49, Napoli, 80131, Italy; 2 Italian Malaria Network - Centro Interuniversitario di Ricerca Sulla Malaria (CIRM) Department of Experimental Medicine and Biochemical Science, Via Del Giochetto, Perugia, Italy; 3 Second University of Naples, Department of Environmental, Biological and Pharmaceutical Sciences and Technologies, Via Vivaldi 43, Caserta, 81100, Italy; 4 Institute of Biostructures and Bioimages (IBB) CNR, Via Mezzocannone 16, Naples, 80134, Italy; 5 Alma Mater Studiorum University of Bologna, Department of Chemistry “G. Ciamician”, Via Selmi 2, Bologna, 40126, Italy; 6 Advanced Accelerator Applications, Via Vivaldi 43, Caserta, 81100, Italy

## Abstract

In the present work we performed a combined experimental and computational study on the interaction of the natural antimalarial endoperoxide plakortin and its synthetic analogue **4a** with heme. Obtained results indicate that the studied compounds produce reactive carbon radical species after being reductively activated by heme. In particular, similarly to artemisinin, the formation of radicals prone to inter-molecular reactions should represent the key event responsible for *Plasmodium* death. To our knowledge this is the first experimental investigation on the reductive activation of simple antimalarial endoperoxides (1,2-dioxanes) by heme and results were compared to the ones previously obtained from the reaction with FeCl_2_. The obtained experimental data and the calculated molecular interaction models represent crucial tools for the rational optimization of our promising class of low-cost synthetic antimalarial endoperoxides.

Malaria, an infectious disease caused by protozoans belonging to the genus *Plasmodium*, continues to affect a large part of the world population, with a special incidence in the sub-Saharan Africa. Indeed, approximately 88% of malaria cases and 90% of malaria deaths occurred in the WHO African Region, with children aged under 5 years and pregnant women most severely affected^1^. Despite the introduction of artemisinin(**1**)-based combination therapies (ACTs) and the mass use of insecticide treated mosquito net (ITN), the pace of decrease in estimated malaria mortality rates slowed down^1^. Indeed, millions of people at risk of malaria still do not have access to interventions such as an ITN, indoor residual spraying (IRS), diagnostic testing, and ACTs. In addition, the emergence of artemisinin-resistant *Plasmodium falciparum (Pf*) strains is raising severe concerns^1^ and the rate at which resistance is growing outpaces the development of new effective and low-cost antimalarials^2^. In the course of a search for new antimalarial lead compounds, we reported that plakortin (**2**) and dihydroplakortin (**3**) (Fig. 1), simple endoperoxide-containing polyketides isolated from the Caribbean sponge *Plakortis simplex*^3^,^4^,^5^, show significant *in vitro* activity against chloroquine (CQ)-resistant strains of *Pf* (i.e., IC_50_ 0.4 μM), promising *in vivo* activity (*P. berghei* infected mice), and no observed toxicity^3^,^4^,^6^.

The preparation of several plakortin semi-synthetic derivatives^7^ allowed the development of a first set of structure-activity relationships (SARs), which strongly suggested that the antimalarial mechanism of action of plakortins, similarly to that of **1**^8^,^9^,^10^,^11^, could include a Fe^II^-induced reductive activation which generates toxic carbon radicals thus leading to the death of the parasite. By using an integrated computational and experimental (FeCl_2_) model, we therefore investigated the Fe^II^-induced reactivity of plakortins^12^. Results confirmed that **2** and **3** react with Fe^II^ undergoing a dissociative electron transfer (DET) of the endoperoxide bond and forming an oxygen-centered radical at O1, which is simultaneously transferred to C10 carbon of **2** or to C13 carbon of **3**, by means of an intra-molecular radical shift mechanism. The resulting carbon radicals were supposed to represent the toxic species able to alkylate biological molecules and to unbalance the redox *Plasmodium* environment. Further SAR studies performed on additional plakortin analogues^13^,^14^,^15^ supported the above mechanism of action. Thus, we set up a low cost Mn^III^-mediated synthesis of a new series of affordable antimalarial endoperoxides, designed on the basis of the plakortin pharmacophore model and characterized by the 3-methoxy-1,2-dioxane scaffold (**4**; Fig. 1)^16^. The new synthetic derivatives showed *in vitro* antimalarial activity on *Pf* CQ-resistant strains comparable to that of the natural leads. Computational and SAR studies performed on the new series of synthetic 1,2-dioxane derivatives indicated that, as in the case of plakortins, the antimalarial activity is related to their ability to react with Fe^II^ generating carbon centered radicals (in this case at C3 and/or at C6 alkyl chain)^16^,^17^,^18^,^19^. Nevertheless, the reaction of **4a** with FeCl_2_ did not evidence any product whose formation could be related to the antimalarial activity^16^.

A recent analysis of the mechanism of action of **1**^20^ showed that free heme-Fe^II^, rather than free Fe^II^, is predominantly responsible for its reductive activation, in agreement with the observed heme-Fe^II^-mediated reactivity of **1** in *in vitro* models^21^,^22^,^23^,^24^,^25^,^26^,^27^,^28^,^29^,^30^. By contrast, neither plakortins nor their synthetic analogues, have been ever investigated for their heme-Fe^II^-mediated reactivity. In order to fulfill this gap and put the base for a rational optimization of this new and promising class of low-cost antimalarials, in the present work we investigated for the first time the interaction of **2** and one of its active synthetic analog (**4a**; Fig. 1) with heme-Fe^II^ chloride dimethyl ester (hereafter heme-Fe^II^-Cl), by NMR spectroscopy and MS analyses. Obtained data are discussed in the frame of our previous results^12^,^13^,^14^,^15^,^16^,^17^,^18^,^19^ and a molecular interaction model of heme-Fe^II^ in complex with the studied ligands was generated.

## Results

### Reaction of heme-Fe^II^-Cl with plakortin 2

NMR was selected as method of choice for the initial characterization of the interaction between **2** and heme. In particular, as shown in Fig. 2, **2** (3 equiv.) was incubated with Fe^III^ protoporphyrin IX dimethyl ester chloride (hemin-Fe^III^-Cl, 1 equiv., **5**) in presence of a large excess of the reducing agent 2,3-dimethylhydroquinone (DMHQ, 10 equiv., **6**), according to the procedure described by Meunier and coworkers for **1**^22^.

The progress of the reaction was closely monitored in real-time in the NMR tube by acquiring a series of mono-dimensional (1D) ^1^H spectra. As can be seen in Supplementary Figure S1, the resonance at 6.53 ppm, assigned to the aromatic protons of **6**, decreased in intensity, while that at 6.72 ppm, the corresponding protons of its oxidized quinone form, increased in intensity. The oxidation of **6**, not observed in the presence of the sole **2** (data not shown), indicates, as expected, the concomitant reduction of hemin-Fe^III^ to heme-Fe^II^. Interestingly, the analysis of the spectra revealed also that the signals of **2** changed over time. In particular, upon the addition of the reducing agent, some signals decreased in intensity and vanished almost completely in ~15 h, whereas other ones exhibited significant broadening (Fig. 2b). These changes were not observed for **2** in the presence of the sole hemin-Fe^III^Cl or reducing agent (data not shown). In particular, the double bond proton signals at 5.38 (H_10_) and 5.10 (H_9_) ppm, the endoperoxide oxymethine signal (H_3_ δ = 4.49 ppm), and signals of other protons surrounding the endoperoxide ring (H_15_ δ = 1.37, H_18_ δ = 3.05) (Fig. 2b) virtually disappeared, suggesting a substantial modification (likely a ring opening) of the endoperoxide skeleton. The broadening of **2** signals, i.e. the methyl groups at around 0.9 ppm (starred H_12_, H_14_ and H_17_ peaks in Fig. 2b), could be due to the formation of an adduct between **2** and hemin-Fe^III^, as a consequence of the presence of paramagnetic Fe^III^.

To gain insight on the reaction products, 2D ^1^H-^13^C HSQC NMR spectra were also acquired. From the spectra analysis, one of the products identified was the chlorinated compound **9** (Fig. 3).

This product, confirmed by MS spectrum, as indicated by the presence of an ion peak at *m*/*z* 349.24, was obtained also when **2** was allowed to react with FeCl_2_^12^. We can then hypothesize that **9** is formed by interaction of **2** with heme-Fe^II^-Cl following the reaction mechanism depicted in Fig. 3.

The one-electron reduction of the peroxide function yields an oxygen radical which is promptly transferred to C10 by an intramolecular rearrangement (1,5-radical shift; **8**, Fig. 3). Once this radical is formed, it reacts with a chlorine atom, possibly linked to the hemin-Fe^III^-Cl, leading to compound **9**. However, while the reaction with FeCl_2_ yielded a mixture of two C10 epimers, in the case of heme only the *10 S* stereoisomer was formed. Indeed, 2D NMR spectra revealed the presence of a single set of characteristic proton and carbon signals, e.g. those at positions 10 (δ_H_ = 3.98, δ_C_ = 65.2) and 9 (δ_H_ = 3.89, δ_C_ = 79.2), assigned by comparison with literature data^12^,^31^ (Supplementary Figure S2).

Additional reaction products of **2** with heme were observed by NMR and MS spectra, which could not be fully characterized. Among them we could identify: (i) a reduced open form of **2** containing two –OH groups at C3 and C6 (Supplementary Figure S2); (ii) an adduct formed by a modified **2** and hemin, as indicated by the presence in the MS spectrum of an ion at m/z 957.59, corresponding to the sum of the masses of hemin and **2** plus one mass unit. Finally, hemin signals, both in the mass (m/z 644.25) and in the NMR spectra (Supplementary Figure S3), indicate that most of hemin remains unmodified upon reaction. Conversely, no MS signals attributable to intact **2** are observed, evidencing that all **2** reacted.

### Reaction of heme-Fe^II^-Cl with the 1,2-dioxane 4a

**Figure 1:**
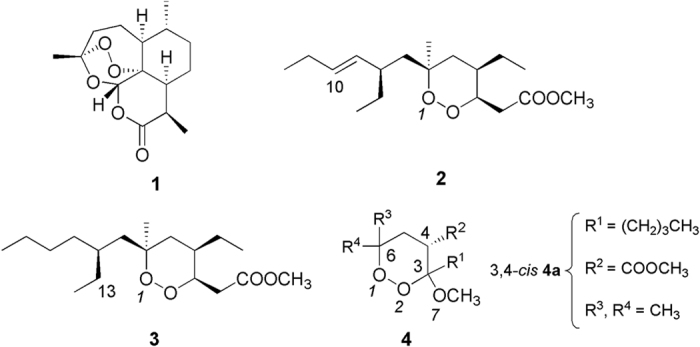
Artemisinin (1), plakortin (2), dihydroplakortin (3), and general structure of 3-methoxy-1,2-dioxane synthetic analogues (4). 3,4-*cis* indicates that **4a** displays the OCH_3_ at C3 and the COOCH_3_ at C4 in a *cis* orientation.

In order to test the generality of the mechanism of action of 3-methoxy-1,2- dioxane scaffold^16^,^17^,^18^,^19^, we selected the active compound **4a** (Fig. 1), a recently developed synthetic analogue of **2**, to reproduce the experiment of interaction with heme-Fe^II^-Cl.

Similarly to **2**, **4a** (3 equiv.) was allowed to react with **5** (1 equiv.) in the presence of the reducing agent **6** (10 equiv.). The progress of the reaction was closely monitored in real-time in the NMR tube by acquiring a series of 1D ^1^H NMR spectra, following peculiar methyl protons of the reagent (H_7_, H_8_, H_14_ and H_18_, Fig. 4).

The NMR analysis indicated that: (i) a fast reaction occurs immediately after the addition of the reducing agent, as shown by the reduced NMR signal intensities already after 10 min; (ii) NMR signals of **4a** (e.g. methyl resonances) decreased at about 50% after 30 min and disappeared almost completely after 6 h, indicating the completeness of the reaction at this time; (iii) simultaneously to the disappearance of the signals of **4a**, several new signals, ascribable to reaction products, appeared in the NMR spectrum (Fig. 5).

A detailed 2D NMR analysis, performed by ^1^H-^1^H TOCSY, ^1^H-^13^C HSQC, ^1^H-^13^C HMBC spectra registered on the reaction mixture, allowed us to identify the structure of several reaction products (Fig. 6; Supplementary Figures S4–S9).

Among them, **13**, **14**, **15**, and **17** were also identified by MS analysis, whereas **10**, **11**, **12**, and **16** could not be detected in the conditions used because of their volatility and low molecular weight. In addition, as for **2**, a signal corresponding to the molecular ion of hemin-Fe^III^ was observed. This finding is in agreement with the NMR analysis, since the hemin signals remain mainly unmodified at the end of the reaction (data not shown).

The formation of the products reported in Fig. 6 can be rationalized by assuming the reductive activation of **4a** by heme-Fe^II^, generated *in situ* by the reducing agent **6**, the homolytic cleavage of the peroxide bond and the formation of the two different alkoxy radicals, either on O1 (pathway a) or on O2 (pathway b) (Fig. 7). The subsequent homolytic cleavage of an adjacent C-C bond (beta scission) can give rise to two different alkyl carbon centered radicals, namely **18** from pathway a, and **19** from pathway b. At this step of the reaction, products **12** and **10** can be formed in the pathway a and b, respectively. In pathway a, the primary C5 centered radical **18** undergoes a classic carbon-carbon bond cleavage with oxidation of O2 and reduction of Fe^III^ to Fe^II^. These intra-molecular rearrangements can lead to the formation of the products **10** and **11**. In pathway b, the α-carbonyl radical **19** can provide the covalent adduct **14** with heme. NMR assignment of **14** signals revealed the presence of the enol form of the carbonyl group (Supplementary Figure S7) as a consequence of its participation in heme conjugation.

Finally, products **13**, **15**, **16**, and **17** are likely formed as a result of different evolutions of the O2 centered radical (pathway b). In particular, the formation of compounds **13**, **15**, and **16** can be rationalized through the following mechanism: a butyl radical is ejected via α-cleavage forming first a methyl ester intermediate bound to the heme by the O1, which is then protonated to **13** with release of heme. Compound **13** then undergoes spontaneously lactonization to **15**. Finally, we hypothesize that the butyl radical undergoes aerobic oxidation to butanal (**16**) under our experimental conditions^32^.

Remarkably, we also identified the chlorinated product **17**. A rationale for the formation of **17** could involve an intra-molecular 1,4-H shift reaction between the endoperoxide oxygen O2 and the C3 butyl chain that leads to the formation of a carbon centered radical on the alkyl chain eventually quenched by a chlorine atom, thus following a reaction pathway similar to that reported for the formation of **9**.

### Molecular modeling

Docking studies on **4a** in complex with heme-Fe^II^ were performed by using a Monte Carlo/Simulated Annealing (SA) based protocol^12^,^33^. In order to enhance the variance of the resulting complexes, two conformers of **4a** were used as starting structures, one presenting intra-molecular distances suitable for the 1,4-H shift and the other presenting intra-molecular distances suitable for the 1,5-H shift (Supplementary Figure S10). Then, following the principles of statistical mechanics, the Monte Carlo-minimization approach randomly generated a total of 19 complexes which were then subjected to a molecular dynamics SA protocol. This included a dynamics run divided in multiple stages, during which the temperature of the system is linearly decreased from 500 K to 300 K. To ensure that the results obtained are independent from the starting geometries, during all calculations the conformational space of the ligand and the target are explored and all rotatable bonds are left free to move. The resulting complexes are subjected to a final optimization without any restraint (see the Methods Section for further details on the computational procedure). All obtained complexes (Supplementary Figure S11) were ranked by their conformational energy (Supplementary Table S1), the lowest energy complex was selected and then subjected to unconstrained DFT full optimization using B3LYP hybrid exchange-correlation functional^34^,^35^ and LanL2DZ basis set^36^,^37^,^38^. A DFT full optimization was also performed on the previously reported^12^ **2**-heme-Fe^II^ complex, obtained applying the same docking procedure herein reported for **4a**. The resulting **2**- and **4a**-heme-Fe^II^ docked complexes are reported in Fig. 8.

It is noteworthy that, comparing the structure of the two starting complexes with those obtained after DFT full optimization (Supplementary Figure S12), a significant change in ligand-heme orientation and C3 (**2**) or C4 (**4a**) chain conformation can be observed, while overall the conformation of C6 (**2**) or C3 (**4a**) alkyl chain is preserved. Interestingly, the relative orientation of the C6 (**2**) or C3 (**4a**) substituent with respect to the endoperoxide function resulted suitable for a 1,5- or 1,4-radical shift from O1 (**2**) or O2 (**4a**), respectively, although in the case of **4a** the docking procedure started from the conformer presenting distances suitable for 1,5-radical shift.

As showed in Fig. 8a, the DFT optimized **2**-heme-Fe^II^ complex evidenced the ability of **2** to interact with Fe^II^ by O1 and O2 (Fe^II^ -O1 = 2.40 Å and Fe^II^ -O2 = 2.98 Å) and, at the same time, to place the double bond close to O1, with C9 correctly positioned for the putative 1,5-radical shift from O1 (O1-C9 = 3.38 Å) (Fig. 3). On the other hand, in the resulting **4a**-heme-Fe^II^ complex (Fig. 8b) the methoxy oxygen atom (O7) drives the interaction with the metal (Fe^II^-O7 distance = 2.28 Å), by consequence, **4a** presents a different peroxide-Fe^II^ coordination mode - including O7, O1, and O2 - from that resulting in the **2**-heme-Fe^II^ complex (Fig. 8b *vs* 8a).

According to our previous DFT calculations^16^, the O7/O1/O2 coordination allows **4a** to form the oxygen radical on both O1 and O2 (Fe^II^ -O1 = 3.67 Å and Fe^II^-O2 = 3.41 Å), although only the O2 radical could be then transferred to a C3 alkyl chain carbon. In the DFT optimized complex the positioning of the C3 butyl chain is indeed optimal for an intra-molecular 1,4-H shift from O2 (O2-H_C10_ = 2.69 Å), and this is true not only for the lowest energy docked complex (Fig. 8b), but also considering all the low energy complexes generated by the docking procedure (Supplementary Figure S11 and Table S1). The stability of the obtained **4a**-heme-Fe^II^ complex was further tested by performing a 50 ns Molecular Dynamics (MD) calculation at 298 K (equilibration phase = 20 ns, production phase = 30 ns; see the Methods Section for further details on the computational procedure) (Supplementary Figure S13; Supplementary Table S2). All complexes obtained during the MD production phase presented a distance suitable for the intra-molecular 1,4 H-shift with calculated non-bond interaction energies ranging between -28 kcal/mol and -51 kcal/mol.

In summary, the resulting bioactive conformations of **2** and **4a** can account for the experimentally derived intra-molecular radical shifts described in Figs 3 and 7, as well as, for the previously observed SARs of this class of this simple antimalarial endoperoxides (1,2-dioxanes)^13^,^14^,^15^,^16^,^17^,^18^,^19^. Accordingly, the calculated complexes represent reliable molecular models which can be used, together with the new experimentally obtained data, for future structure optimization.

## Discussion

The results of the present investigation supply new evidences to the antimalarial mechanism of action we previously proposed for plakortins and their synthetic analogues^12^,^13^,^14^,^15^,^16^,^17^,^18^,^19^. Upon interaction with heme-Fe^II^-Cl, **2** and **4a** undergo the reductive cleavage of the endoperoxide bond through a DET mechanism, with the formation of an oxygen-centered radical which quickly evolves to carbon-centered toxic radicals, the putative “bioactive species” that kill the parasite (Figs 3 and 7). In particular, after the reaction of **2** with heme-Fe^II^-Cl, the chlorinated compound **9** and a covalent **2**-heme adduct were detected as main products, demonstrating the propensity of the formed carbon radicals to promote inter-molecular reactions. These reactions represent a crucial issue for antimalarial activity; strong evidences have been, indeed, recently provided^20^ that heme-Fe^II^-activated **1** kills the malaria parasite through a promiscuous targeting mechanism including the covalent binding to 124 protein targets. The observation of **2**-heme adduct signals after a few days witnesses it is not a short-lived species in these reaction conditions, the same was observed for the **4a**-heme adduct discussed below. The formation of **9**, also observed upon FeCl_2_ reaction^12^, results by an inter-molecular reaction of the C10-centered radical species, obtained, in turn, through an intra-molecular radical shift from O1 to the C9-C10 double bond (Figs 3 and 8a). The precise configuration obtained at C10, not observed in the reaction with FeCl_2_, suggests that complexation of **2** with heme makes the two diastereotopic faces of the C10 centered radical differently accessible to the approaching chlorine atom.

This hypothesis is supported by the calculated **2**-Fe^II^-heme interaction model, showing that just the Si face of the C9-C10 bond is accessible to inter-molecular reactions (Fig. 8a). If we hypothesize that the chlorine atom approaches C10 by using this face only the 10 S stereoisomer would be formed, as indeed found in **9**. A higher steric accessibility of both faces is likely to characterize, on the contrary, the C10-centered radical when FeCl_2_ is used^12^.

**Figure 2:**
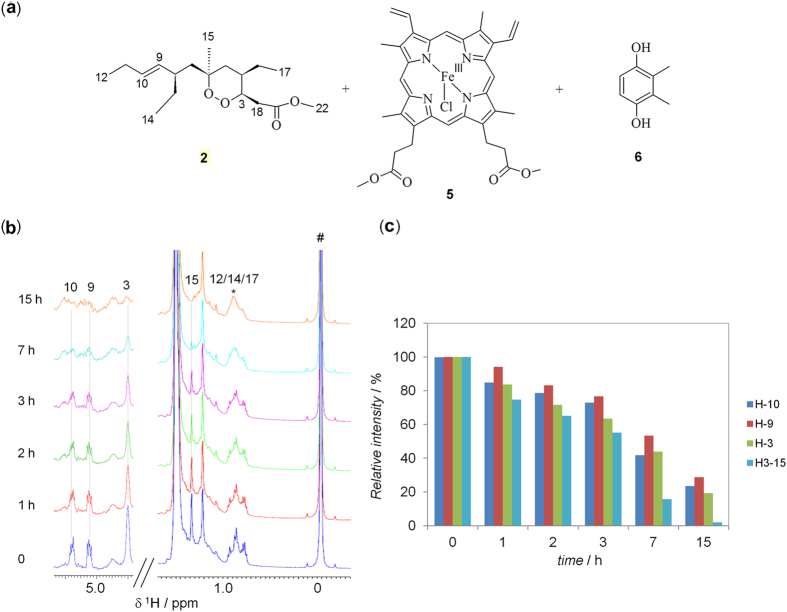
Reaction between plakortin (3 equiv., 2) and hemin-Fe^III^-Cl (1 equiv., 5) in presence of the reducing agent, 2,3-dimethylhydroquinone (10 equiv., 6) in deuterated chloroform at 25 °C. (**a**) Chemical structure of the reagents. (**b**) ^1^H NMR time-course of the reaction. Proton resonances of **2**, decreasing in intensity or broadening upon reaction, are indicated with dotted lines or an asterisk, respectively, and numbered as reported in the structure in (**a**). Different vertical scales were used in the two sections of the ^1^H NMR spectra. The peak indicated with # is from tetramethylsilane (TMS). (**c**) Bar graphs of the percentage of **2** signal intensity, relative to the initial value (t = 0) of 100%.

In the case of **4a**, a faster reaction with heme was observed (Figs 2b vs. 4b). However, the intra-molecular 1,4-radical shift reaction, involving the endoperoxide function and the C3 butyl chain (Figs 7 and 8b), competes with several carbon-carbon bond cleavage reactions. This could explain the lower activity of **4a** with respect to **2** despite its higher reactivity. In particular, as predicted by our calculations, the Fe^II^ coordination mode of **4a** allows the formation of the oxygen radicals on both O1 and O2 producing not only the chlorinated product **17**, resulting from the intra-molecular 1,4-hydrogen shift reaction, but also compounds **10–16**, obtained by the carbon-carbon bond cleavage reactions (Fig. 7). Nevertheless, similarly to what already observed by Posner *et al*. for **1**^10^,^11^, the lack of any SAR indicating that the observed carbon-carbon cleavage reactions are responsible for the antimalarial activity, suggests that these reactions could compete with the formation of the toxic radical species by producing carbon radicals which promptly evolve into neutral species through intra-molecular rearrangements (Fig. 7). An important exception is represented by the radical species **19** (generated by the radical shift from O2 to C4), which is responsible for the formation of the heme-adduct **14** (Fig. 7). Interestingly, a pharmacodynamic role of the C4 substituent was recently observed by us when replacing the ester group of **4a** with an amino group, and a possible involvement in the putative toxic radical formation/propagation has been evoked^19^. The C4 substitution determined, indeed, a new and more potent series of analogues showing peculiar SARs compared to the previous derivatives, being able to increase the antimalarial activity independently from the presence and position of the C3/C6 butyl chain(s)^19^. Thus, the newly obtained results provide a possible explanation for such behavior putting the rational bases for the development of new analogues.

**Figure 3:**
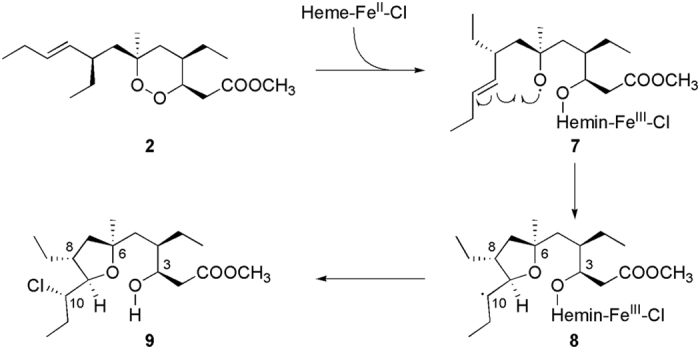
Mechanism proposed for the formation of compound 9 upon reaction of plakortin (2) with heme-Fe^II^-Cl.

**Figure 4:**
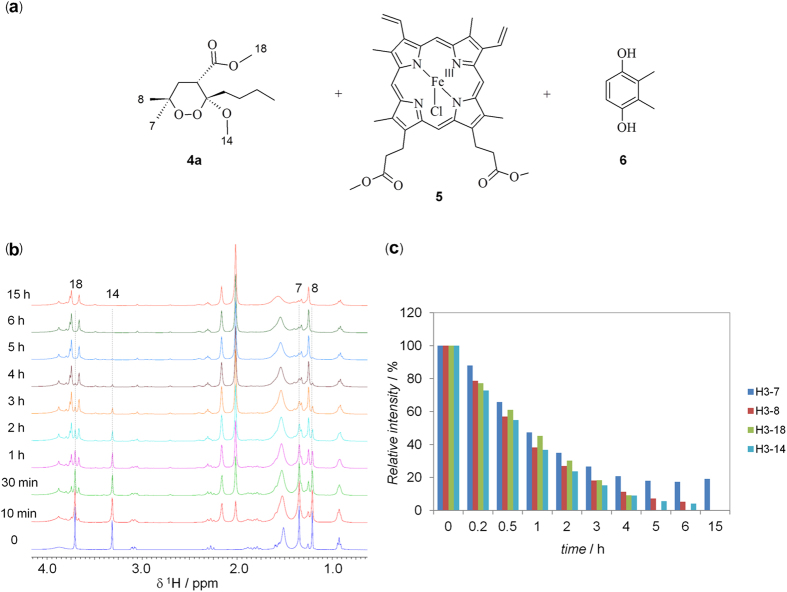
(**a**) Chemical structure of the reagents. (**b**) ^1^H NMR time-course of the reaction. Some proton resonances of **4a**, decreasing in intensity, are indicated with dotted lines and numbered as reported in the structure in (A). (**c**) Bar graphs of the percentage of **4a** signal intensity, relative to the initial value (t = 0) of 100%.

**Figure 5:**
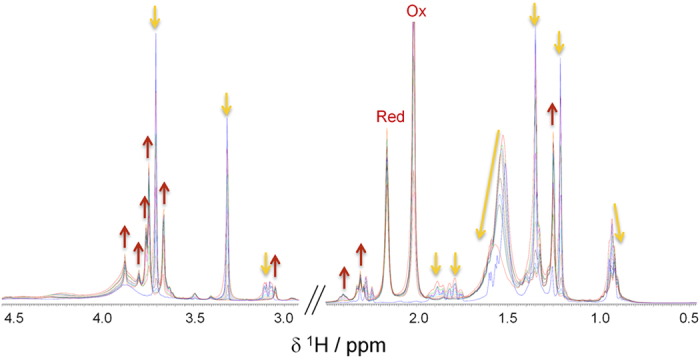
Products (up red arrows) and reagents (down yellow arrows) ^1^H NMR signals upon reaction between 3-methoxy-1,2-dioxane derivative (**4a**) and hemin (**5**) in presence of the reducing agent, 2,3-dimethylhydroquinone (**6**).

**Figure 6:**
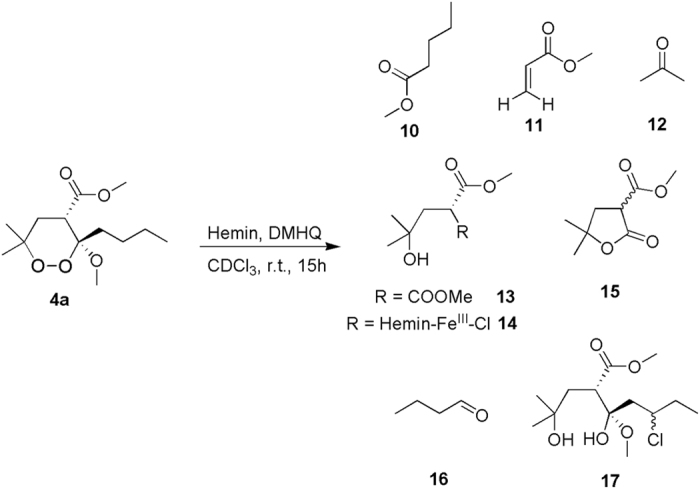
Products obtained upon reaction of 3-methoxy-1,2-dioxane derivative (4a) with hemin and DMHQ.

**Figure 7:**
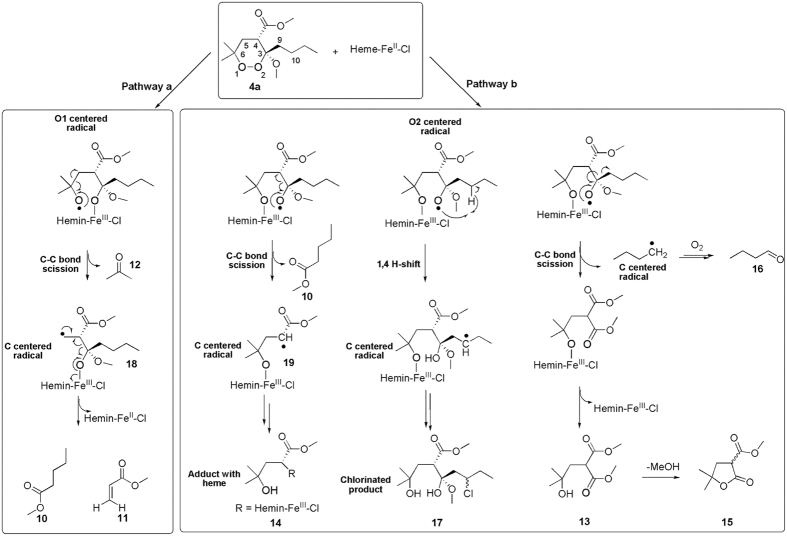
Proposed reaction mechanism of the synthetic analog 4a with heme-Fe^II^-Cl.

**Figure 8:**
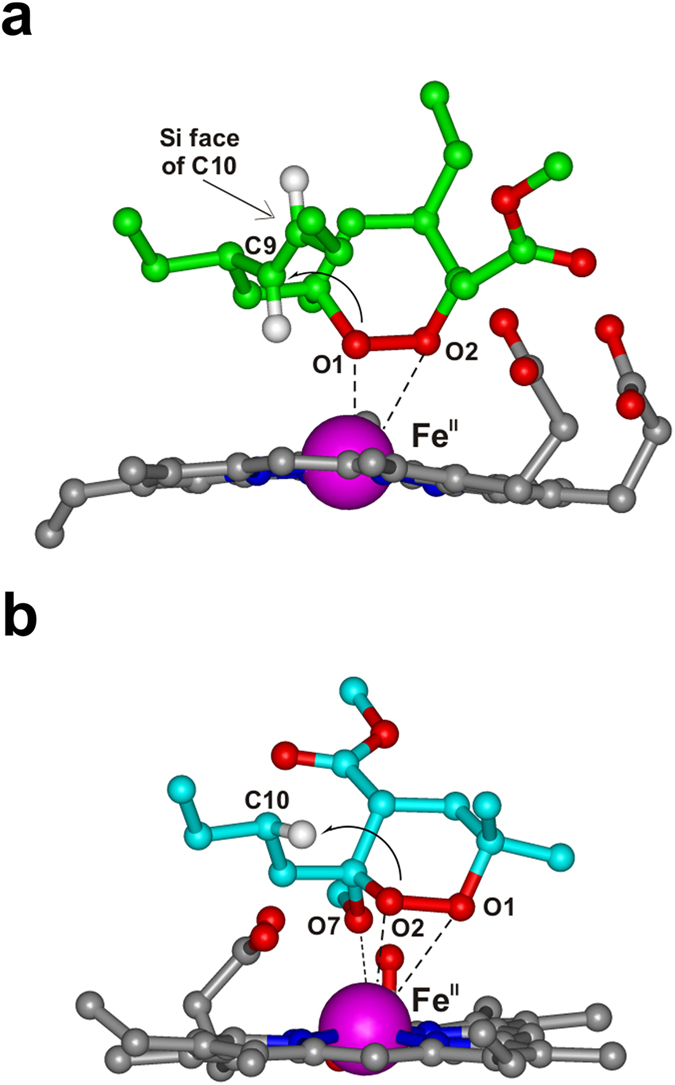
DFT optimized structure of 2 (carbon green; a) and **4a** (carbon cyan; b) in complex with Fe^II^-heme (grey). Heteroatoms are coloured by atom type (O = red; N = blue; Fe = magenta; H = white). Hydrogens are omitted for sake of clarity with the exception of those involved in the putative intra-molecular radical shift. The vdW volume of Fe^II^ is scaled by 50% for clarity of presentation. Atoms coordinating Fe^II^ (dashed line), as well as, those involved in the putative radical shift are labelled. In (a) the C10 diastereotopic face available for inter-molecular reactions is indicated.

The comparison of the present results with those previously obtained by testing the heme-alkylating ability of **1** and synthetic antimalarial trioxanes and trioxolanes^21^,^22^,^23^,^24^,^25^,^26^,^27^,^28^,^29^,^30^ supports the hypothesis that the trend to form covalent adducts with heme is related to the antimalarial potency. However, the formation of chlorinated products **9** (Fig. 3) and **17** (Fig. 7) starting from **2** and **4a**, respectively, reveals that further alkylating radicals are in action, resulting from the shift of the oxygen radical to a carbon of the alkyl chain. We already reported^12^,^13^,^14^,^15^,^16^,^17^,^18^,^19^ that the ability to form such carbon radicals can account for the observed antimalarial potency of **2** and its natural and synthetic derivatives.

It has to be underlined that the putative “bioactive species” of **4a** were determined only when **4a** was allowed to react with heme, as in the present study, and not when we performed the reaction with FeCl_2_^16^. Indeed, in the latter case, we identified as major product only the carbon-carbon cleavage product **15** (Fig. 7). This strongly suggests, as reported for **1**^20^, that heme, rather than free ferrous iron, is predominantly responsible for antimalarial endoperoxide “bio-activation”, showing that the investigation of the interaction of natural or synthetic compounds with heme by using NMR spectroscopy and MS analyses, could represent an efficient screening method for the evaluation of their antimalarial activity.

During the erythrocyte stage of the *Plasmodium* the (bio)activation of the endoperoxide function likely occurs by the heme-Fe^II^ derived from hemoglobin digestion. The reduction of the endoperoxide bond by agents, such as free heme-Fe^II^, which are not present in normal host cells, represents a key issue for the selective toxicity of these compounds, and, hence, for their potential development as antimalarial drugs. Remarkably, both **2**^3^,^4^ and **4a**^16^ showed no toxicity against human cells and a recent biochemical investigation demonstrated that **2** induces ROS production and cause cellular damages only in infected human erythrocytes^39^.

Altogether, our results indicate that **2**, **4a**, and their analogues, similarly to **1**^20^, do not interact with a specific protein target but, rather, produce toxic radical species after being reductively activated by heme-Fe^II^. The formation of radicals prone to intermolecular reactions, should represent the key event responsible for a reaction cascade eventually leading to *Plasmodium* death. Such varied and unspecific mechanism of action is more difficult to be bypassed by parasite resistance than a mechanism of action based on the interaction with a specific protein target.

## Conclusions

We have herein presented the first experimental investigation on the interaction of simple antimalarial endoperoxides (1,2- dioxanes) with heme. Obtained results provided new information with respect to the previously reported reaction with FeCl_2_. The knowledge of further details on the molecular mechanism of action of plakortins and their synthetic analogues constitutes an issue of key importance to continue the investigation on antimalarial endoperoxides. Indeed, only this knowledge can lead to a more detailed comprehension of the structural features responsible for antimalarial activity and their rational optimization. For example, more efficient substituents patterns on the 1,2-dioxane ring could be devised, in order to exploit all possible reactions for the generation of various toxic radical species.

## Methods

### General chemical procedures

NMR spectra were recorded at 25 °C using Inova 400 MHz and Inova 600 MHz, equipped with a cryogenic probe optimized for ^1^H detection, spectrometers (Varian Inc., Palo Alto, CA, USA). Chemical shifts were referenced to internal Tetramethylsilane (TMS). Reactions were monitored by mono-dimensional (1D) ^1^H NMR spectra. Products were characterized by 1D and bi-dimensional (2D) homo- and hetero-nuclear spectra. Homo-nulclear ^1^H-^1^H scalar correlations were determined by TOCSY (Total Correlation Spectroscopy) experiments with mixing time of 70 ms. One-bond heteronuclear ^1^H-^13^C correlations were determined by HSQC (Heteronuclear Single Quantum Coherence) experiments. Two and three-bond heteronuclear ^1^H-^13^C correlations were determined by HMBC (Heteronuclear Multiple Bond Correlation) experiments with gradients of refs 2 and 3 J from 5 to 15 Hz. Low- and high-resolution ESI-MS spectra were performed on a LTQ Orbitrap XL (ThermoScientific) mass spectrometer.

### Material

Ferriprotoporphyrin IX dimethyl ester chloride [hemin-Fe^III^-Cl, **5**] and 2,3-dimethylhydroquinone (DMHQ, **6**) are from commercial sources (Livchem Logistics GmbH and Carlo Erba Reagents, respectively). Plakortin (**2**) was obtained as previously reported^40^. 3-methoxy-1,2-dioxane derivative (**4a)** was synthesized, purified and characterized as already reported^16^.

### Reaction of heme-Fe^II^-Cl with plakortin 2

Plakortin (**2**, 2.8 mg, 9.0 μmol) was mixed with the hemin-Fe^III^-Cl (**5**, 2.0 mg, 2.9 μmol) in deuterated chloroform (500 μL) in a NMR tube. A 1D ^1^H NMR spectrum of the obtained solution was acquired before the reductant was added. Large spectral width of 31974.42 Hz and short relaxation delay 0.4 s were used to visualize the very low-field and broad signals of high-spin ferric hemin. Then, 2,3-dimethylhydroquinone (**6,** 4.1 mg, 29.7 μmol) was added as solid. The oxygen at the top of the reaction mixture was replaced with nitrogen gas. The reaction was monitored acquiring 1D ^1^H NMR spectra every hour for 15 h. The following product was identified. Compound **9**: ^1^H and ^13^C NMR: see data reported in ref. 12 MS: m/z: 349.24 [M]^+^.

### Reaction of heme-Fe^II^-Cl with synthetic 3-methoxy 1,2-dioxane 4a

Compound **4a** (12.5 mg, 48 μmol) was mixed with the hemin-Fe^III^-Cl (**5**, 10.9 mg, 16 μmol) in deuterated chloroform (500 μL) in a NMR tube. Then, 2,3-dimethylhydroquinone (**6,** 22.1 mg, 160 μmol) was added as solid. The oxygen at the top of the reaction mixture was replaced with nitrogen gas. The reaction was monitored for 15 h, acquiring 1D ^1^H NMR spectra after 10, 30 and 60 min and after every hour. The following products were identified. Compound **10**. ^1^H NMR (600 MHz, CDCl_3_): *δ = *3.66 (s, 3 H; COOMe); 2.31 (t, J = 7.3 Hz, 2 H; H-2); 1.60–1.62 (m, J = 7.3 Hz, 2 H; H-3); 1.35-1.33 (m, J = 7.3 Hz, 2 H; H-4); 0.91 (t, J = 7.3 Hz, 3 H; H-5). ^13^C NMR (600 MHz, CDCl_3_): *δ* = 174.45 (C1); 51.43 (COOMe); 33.89 (C2); 27.09 (C3); 22.27 (C4); 13.81 (C5). In agreement with chemical shifts reported in Spectral Database for Organic Compounds (SDBS) No 2890. Compound **11**. ^1^H NMR (600 MHz, CDCl_3_): *δ *= 6.41 (d, J = 18.6 Hz, 1 H; H-3b); 6.15-6.10 (dd, J = 18.6, 10.8, H-2); 5.82 (d, J = 10.8, 1 H; H-3a); 3.76 (s, 3 H; COOMe). ^13^C NMR (600 MHz, CDCl_3_): *δ = *166.69 (COOMe-1); 130.61 (C3); 128.41 (C2); 51.65 (COOMe). In agreement with chemical shifts reported in SDBS No 1220. Compound **13**. ^1^H NMR (600 MHz, CDCl_3_): *δ = *3.74 (s, 6 H; COOMe); 3.63 (overlapped, 1 H; H-2); 2.16 (overlapped, 2 H; H-3); 1.24 (s, 6 H; H-5 and H-6). ^13^C NMR (600 MHz, CDCl_3_): *δ* = 170.69 (COOMe-1 and -7); 70.11 (C4); 52.65 (COOMe); 47.72 (C2); 41.77 (C3); 29.38 (C5 and C6). MS: m/z: 227.09 [M + Na]^+^ Compound **14** (Enol form). ^1^H NMR (600 MHz, CDCl_3_): *δ* = 2.77 (s, 2 H; H-3); 1.60 (s, 6 H; H-5 and H-6). ^13^C NMR (600 MHz, CDCl_3_): *δ* = 194.20, (C1); *δ* = 81.20 (C4); 46.70 (C3); 29.40 (C5 and C6). MS: m/z: 804.28 [M + 16]^+^ Compound **15**: ^1^H and ^13^C NMR: see data reported in ref. 14a. MS: m/z: 195.06 [M + Na]^+^ Compound **16**. ^1^H NMR (600 MHz, CDCl_3_): *δ* = 9.77 (s, 1 H; H-1); 2.40 (t, 2 H; H-2); 1.68 partially overlapped (m, 2 H; H-3); 0.97 (t, J = 7.3 Hz, 3 H; H-4). ^13^C NMR (600 MHz, CDCl_3_): *δ* = 203.09 (C1); 45.96 (C2); 15.75 (C3); 13.80 (C4). Compound **17**. ^1^H NMR (600 MHz, CDCl_3_): *δ* = 3.70 (s, 3 H; COOMe-12); 3.65 (1 H; H-3); 3.30 (s, 3 H; OMe-13); 3.05 (1 H; H-6); 1.56 (m, 2 H; H-2); 1.32 (s, 6 H; H-9 and H-10); 0.92 (d, 3 H; H-1). All proton signals was overlapped in the 1 H NMR spectrum and were assigned with 2D COSY, HSQC and HMBC spectra. ^13^C NMR (600 MHz, CDCl_3_): *δ = *171.44 (COOMe-11); 102.00 (C5); 70.15 (C8); 51.99 (COOMe-12); 48.53 (OMe-13); 41.77 (C3); 34.85 (C2); 29.24 (C9 and C10). MS: m/z: 319.12 and 321.12 (100:33) [M + Na]^+^. When J values are not indicated, they could not be measured for overlapping.

### Molecular modeling

Molecular modeling calculations were performed on SGI Origin 200 8XR12000 and E4 Server Twin 2X Dual Xeon-5520, equipped with two nodes. Each node: 2X Intel^®^ Xeon^®^ QuadCore E5520-2.26Ghz, 36 GB RAM.

#### Docking Procedure

Docking studies were carried out on **4a** in complex with heme-Fe^II^, using a Monte Carlo-metropolis/SA docking methodology which considers all the systems flexible (Affinity, SA_Docking; Insight 2005, Accelrys, San Diego, CA)^33^. Heme apparent pKa values were calculated by using the ACD/Percepta software^41^. Atomic potentials were assigned by the Heme29.frc^42^ force field while atomic partial charges were assigned by PM7 calculations^43^. Two different starting confomers of **4a** were selected among those previously calculated^16^: the one resulting from DFT calculations in complex with Fe^II^ and the PM7 global minimum conformer. All atoms in the complex were left free to move during the entire docking procedure with the exception of heme pyrrolic carbons, which were tethered to their original position. Non-bond interactions were calculated using the Cell_Multipole method^44^. A Monte Carlo/minimization approach for the random generation of a maximum of 20 **4a**-heme-Fe^II^ complexes was applied. During the first step, starting from the previously obtained roughly docked structures, the ligand was moved by a random combination of translation, rotation, and torsional changes (Flexible_Ligand option, considering all rotatable bonds) to sample both the conformational space of the ligand and its orientation with respect to the heme-Fe^II^ (MxRChange = 1 Å; MxAngChange = 180°). During this step, van der Waals (vdW) and Coulombic terms were scaled to a factor of 0.1 to avoid very severe divergences in the Coulombic and vdW energies. If the energy of a complex structure resulting from random moves of the ligand was higher by the energy tolerance parameter than the energy of the last accepted structure, it was not accepted for minimization. To ensure a wide variance of the input structures to be successively minimized, an energy tolerance value of 10^6^ kcal mol^−1^ from the previous structure has been used. After the energy minimization step (conjugate gradient, 2500 iterations, ε = 80*r), the Metropolis test, at a temperature of 310 K, and a structure similarity check (rms tolerance of 0.3 kcal Å^−1^) were applied to select the 20 acceptable structures. Each subsequent structure was generated from the last accepted structure. All the accepted complexes resulting from the Monte Carlo/minimization approach were subjected to a molecular dynamics simulated annealing protocol, including 5 ps of a dynamic run divided in 50 stages (100 fs each) during which the temperature of the system was linearly decreased from 500 to 300 K (Verlet velocity integrator; time step of 1.0 fs). Molecular dynamics calculations were performed using a constant temperature and constant volume (NVT) statistical ensemble, and the direct velocity scaling as temperature control method (temp window, 10 K). In the first stage, initial velocities were randomly generated from the Boltzmann distribution according to the desired temperature, while during the subsequent stages initial velocities were generated from dynamics restart data. A temperature of 500 K was applied to surmount torsional barriers, thus allowing an unconstrained rearrangement of **4a** and heme-Fe^II^ (initial vdW and Coulombic scale factors of 0.1). Successively temperature was linearly reduced to 300 K in 5 ps, and concurrently the scale factors were similarly increased from their initial values (0.1) to their final values (1.0). Distance restraints were applied between Fe^II^ and the interacting oxygens in order to avoid unrealistic results during the SA procedure (100 kcal/mol/Å). A final round of 10^5^ unconstrained minimization steps (conjugate gradient, ε = 80*r) followed the last dynamics steps, and the minimized structures were saved in a trajectory file. After this procedure, the resulting docked structures were ranked by their conformational energy and classified according to the conformational properties of the ligand (i.e., torsional angles). The complex with the best conformational energy was selected as the complex representing the most probable **4a-**heme-Fe^II^ binding mode.

#### DFT Calculations

The selected **4a**-heme-Fe^II^ complex and the previously calculated^12^ **2**-heme-Fe^II^ complex were subjected to Density Functional Theory (DFT) optimization. DFT calculations were performed with Gaussian 09 program suite^45^ by using B3LYP hybrid exchange correlation functional^34^,^35^ and LanL2DZ basis set^36^,^37^,^38^. Fe^II^ was considered in the quintet spin multiplicity^46^. Complex optimization was carried out without imposing geometric constraints and vibrational analysis was performed at the same level of theory.

#### Molecular dynamics procedure

DFT optimized structure of **4a** in complex with Fe^II^-heme was subjected to a Molecular Dynamics (MD) calculation using Discover_3 module of Insight 2005 (Accelrys Software Inc., San Diego, CA). MD simulation was carried out in two phases: an equilibration phase of 20 ns and a production phase of 30 ns. All MD simulation was performed using a constant temperature and constant volume (NVT) statistical ensemble and mimicking the polarity of a water media (ε = 80*r). Non-bond interactions were calculated using the Group Based method (CUT_OFF: 100). During the entire MD procedure all atoms in the complex were left free to move whereas a tethering restraint was applied on heme pyrrolic carbons, which were tethered to their original position, and distance restraints (2.5 Å–4.0 Å) were applied between Fe^II^ and the interacting oxygens (10 kcal/mol/Å). During the equilibration phase, the **4a**-heme-Fe^II^ complex was subjected to a 20 ns of MD at 298 K using a time step of 1 fs (Verlet velocity integrator). Initial velocities were randomly generated from the Boltzmann distribution according to the initial temperature (T = 10 K) and the direct velocity scaling method was used to control the temperature of the system with the Temperature Difference parameter set to 10 K. After the equilibration period, the production phase was run for 30 ns, using a time step of 0.5 fs with a constant temperature of 298 K (Verlet velocity integrator). Initial velocities were generated from dynamics restart data and the Nosé–Hoover method was used to control the temperature of the system (Q_ratio = 1.0). The coordinates of the system were saved in a trajectory file every 1 ns and the non-bond interaction energies of the corresponding complexes were calculated (vdW and electrostatic energy contribution; CUT_OFF = 100; Insight 2005 Docking module (Accelrys Software Inc., San Diego, CA)).

## Additional Information

**How to cite this article:** Persico, M. *et al*. The interaction of heme with plakortin and a synthetic endoperoxide analogue: new insights into the heme-activated antimalarial mechanism. *Sci. Rep.* **7**, 45485; doi: 10.1038/srep45485 (2017).

**Publisher's note:** Springer Nature remains neutral with regard to jurisdictional claims in published maps and institutional affiliations.

## Supplementary Material

Supplementary Information
