# Supplementary material for: The interaction of heme with plakortin and a synthetic endoperoxide analogue: new insights into the heme-activated antimalarial mechanism
Source: Sci Rep. 2017 Apr 6;7:45485. doi: 10.1038/srep45485 (PMC5382535; doi:10.1038/srep45485)
Supplement: Supplementary Information [file srep45485-s1.pdf]

SUPPORTING INFORMATION FOR

The interaction of heme with plakortin and a synthetic endoperoxide analogue: new insights into the heme-activated antimalarial mechanism

Marco Persico[1,5] Roberto Fattorusso[2,4] Orazio Taglialatela-Scafati[1,5] Giuseppina Chianese[1,5] Ivan de Paola[4] Laura Zaccaro[4] Francesca Rondinelli[1,5] Marco Lombardo[3,5] Arianna Quintavalla[3,5] Claudio Trombini[3,5] Ernesto Fattorusso[1,5,†] Caterina Fattorusso[1,5,]* and Biancamaria Farina[4,5,]*

[1]*University of Naples "Federico II", Department of Pharmacy, Via D. Montesano 49, Napoli, 80131, Italy*

[2]*Second University of Naples, Department of Environmental, Biological and Pharmaceutical Sciences and Technologies, Via Vivaldi 43, Caserta, 81100, Italy*

[3]*Alma Mater Studiorum University of Bologna, Department of Chemistry "G. Ciamician", Via Selmi 2, Bologna, 40126, Italy*

[4]*Institute of Biostructures and Bioimages (IBB) CNR, Via Mezzocannone 16, Naples, 80134, Italy*

[5]*Italian Malaria Network - Centro Interuniversitario di Ricerche Sulla Malaria (CIRM) Department of Experimental Medicine and Biochemical Science, Via Del Giochetto, Perugia, Italy*

[6]*Advanced Accelerator Applications,Via Vivaldi 43, Caserta, 81100, Italy*

*caterina.fattorusso@unina.it;bianca.farina@adacap.com

†*Dedicated to the beloved memory of prof. Ernesto Fattorusso*

Table of Contents:

1. Title, Affiliations, Table of Contents **S1**
2. NMR studies (Figures S1-S3) **S2-S4**
3. NMR spectra used for the assignments of the products obtained by reaction of the compound **4a** with heme (Figures S4-S9) **S5-S10**
4. Molecular docking and dynamics studies (Figures S10-S13 and Tables S1-S2) **S11-S16**

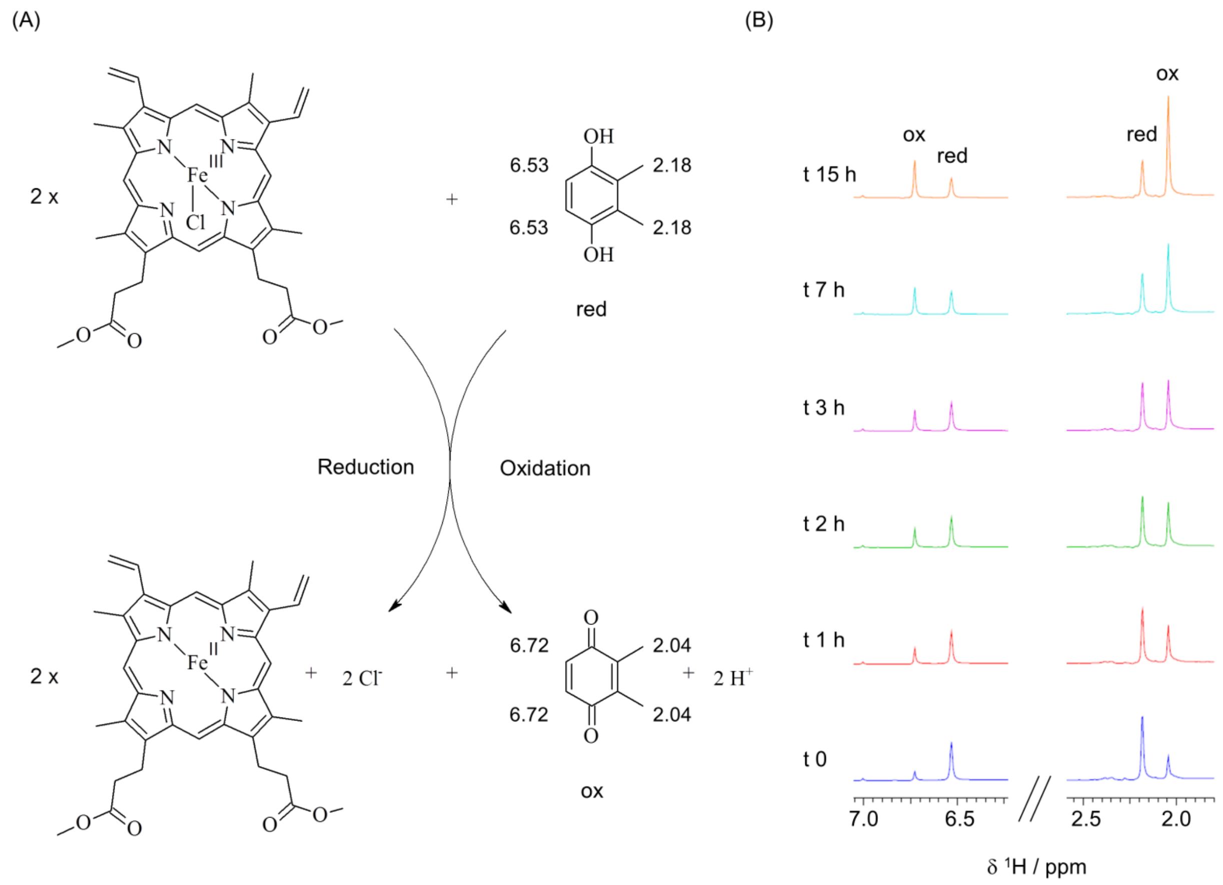

Figure S1. (A) Scheme of the reduction reaction of Fe^{III} protoporphyrin IX dimethyl ester chloride (hemin-Fe^{III}-Cl) to Fe^{II} protoporphyrin IX dimethyl ester chloride (hemin-Fe^{II}-Cl) with 2,3-dimethylhydroquinone (red, 10 eq.) in deuterated chloroform at 25°C. (B) ^{1}H NMR spectra time-course for the reaction proton signals of 2,3-dimethylhydroquinone at 6.53 and 2.18 ppm, labeled as red, decreased in intensity, and, concomitantly, proton signals of the oxidized form (ox) at 6.72 and 2.04 ppm, labeled as ox, increased in intensity. Same vertical scale was used in the two sections of the ^{1}H NMR spectra.

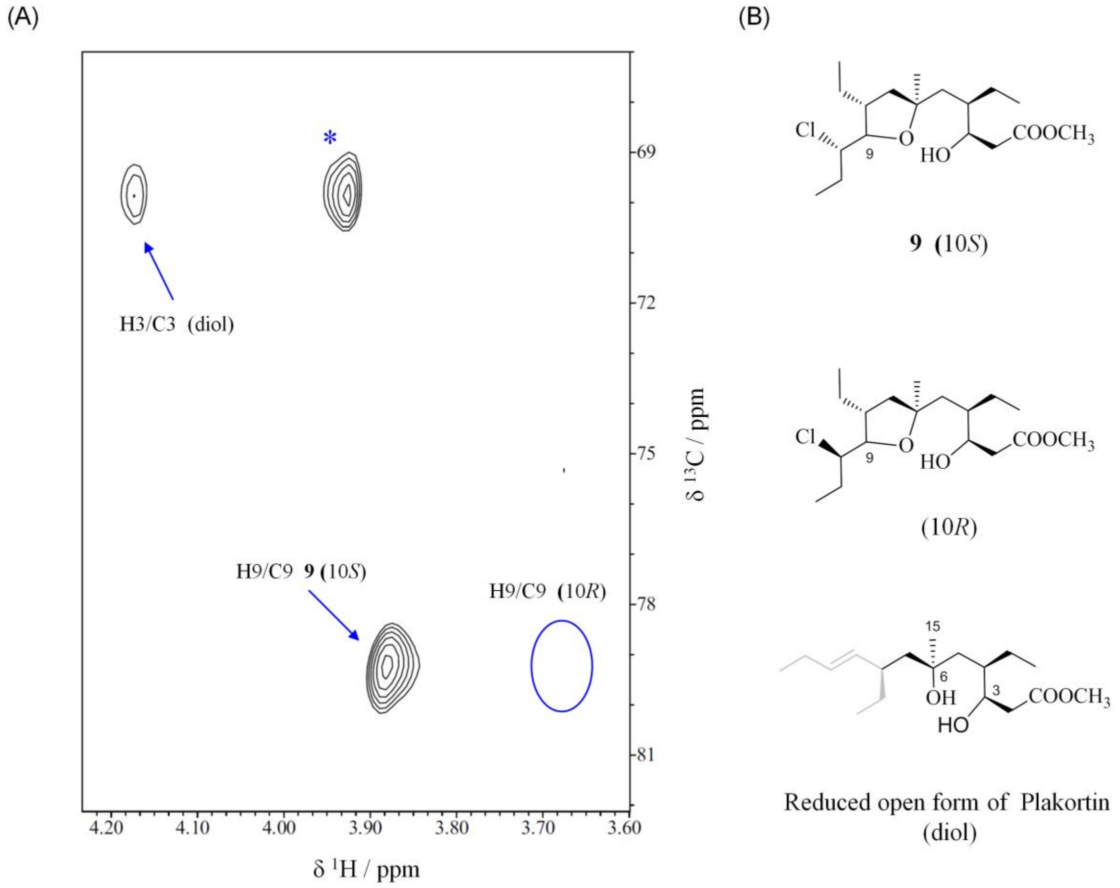

Figure S2. (A) Section of the ^{1}H-^{13}C HSQC spectrum of the reaction mixture [Plakortin (3eq.), hemin (1 eq.) and 2,3-dimethylhydroquinone (10 eq.)] after incubation of 15h. (B) Chemical structure of the product **9** (10*S*), its diasteroisomer (10*R*) and the product characterized by the presence of two hydroxyls in position C3 and C6 (diol). In (A), characteristic proton and carbon signals of identified products (H_9/C9 for 10*S* and H_3/C3 for the diol) are indicated by an arrow. The absence of the H_9/C9 cross-peak (indicated by a circle) of the 10*R*, also known as plakortether C and previously characterized in ref. 31, confirms the formation of only 10*S* diasteroisomer. A not assigned cross-peak is marked by an asterisk.

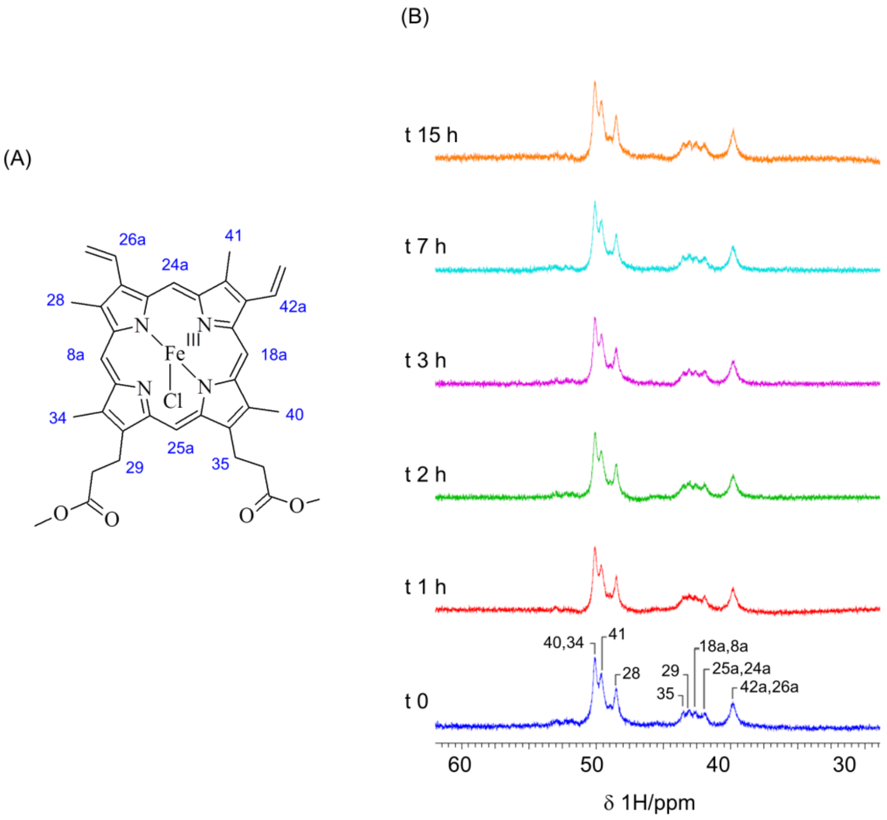

Figure S3. (A) Structure of hemin-Fe^{III}-Cl. (B) Downfield region of the ^{1}H NMR spectra for the reaction between Plakortin (3 eq.) and hemin-Fe^{III}-Cl (1 eq.) in presence of the 2,3-dimethylhydroquinone (10 eq.) in deuterated chloroform at 25°C. Proton signals of hemin-Fe^{III}-Cl are labelled and numbered as reported in (A).

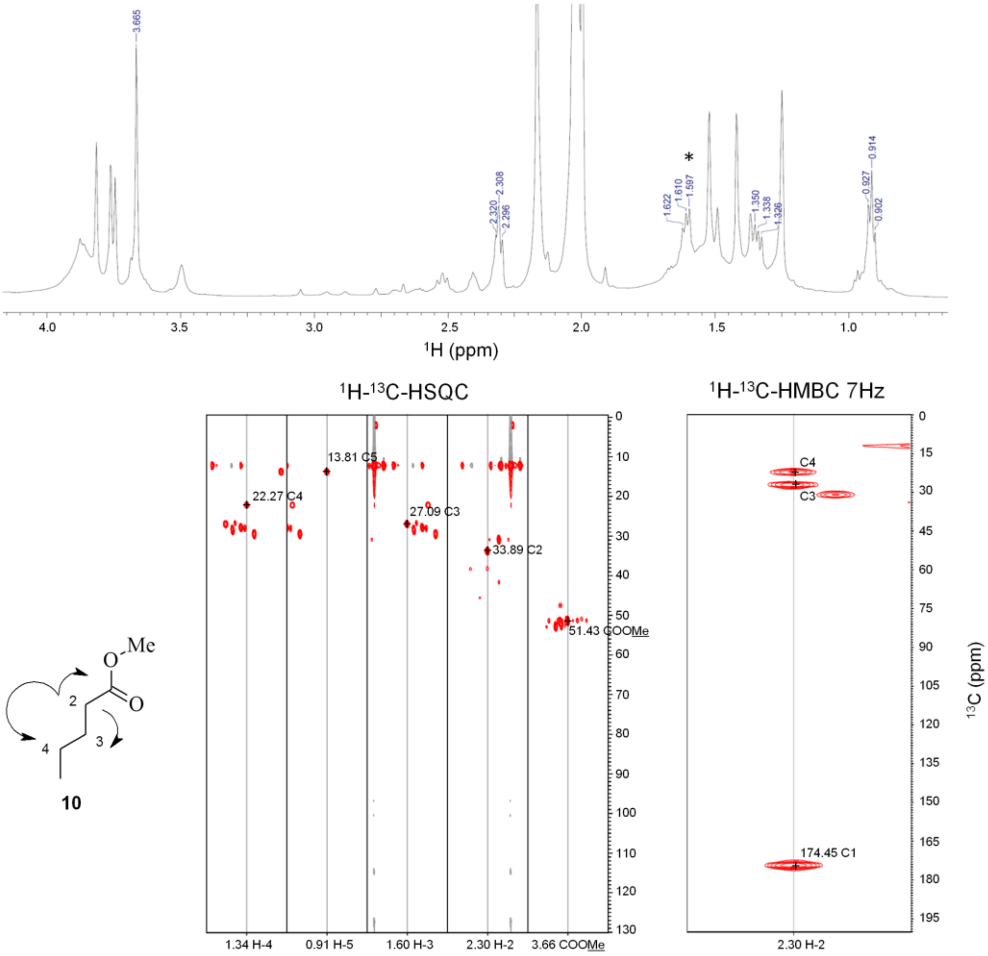

Figure S4. NMR spectra used for the proton and carbon assignments of the compound **10**. For clarity, bi-dimensional HSQC and HMBC spectra are showed as "strip plot". Typically, along the x axis each strip is centred to the chemical shift value of an individual peak of the compound. Key HMBC cross-peaks detected are indicated on the structure. Overlapped signals are marked by an asterisk.

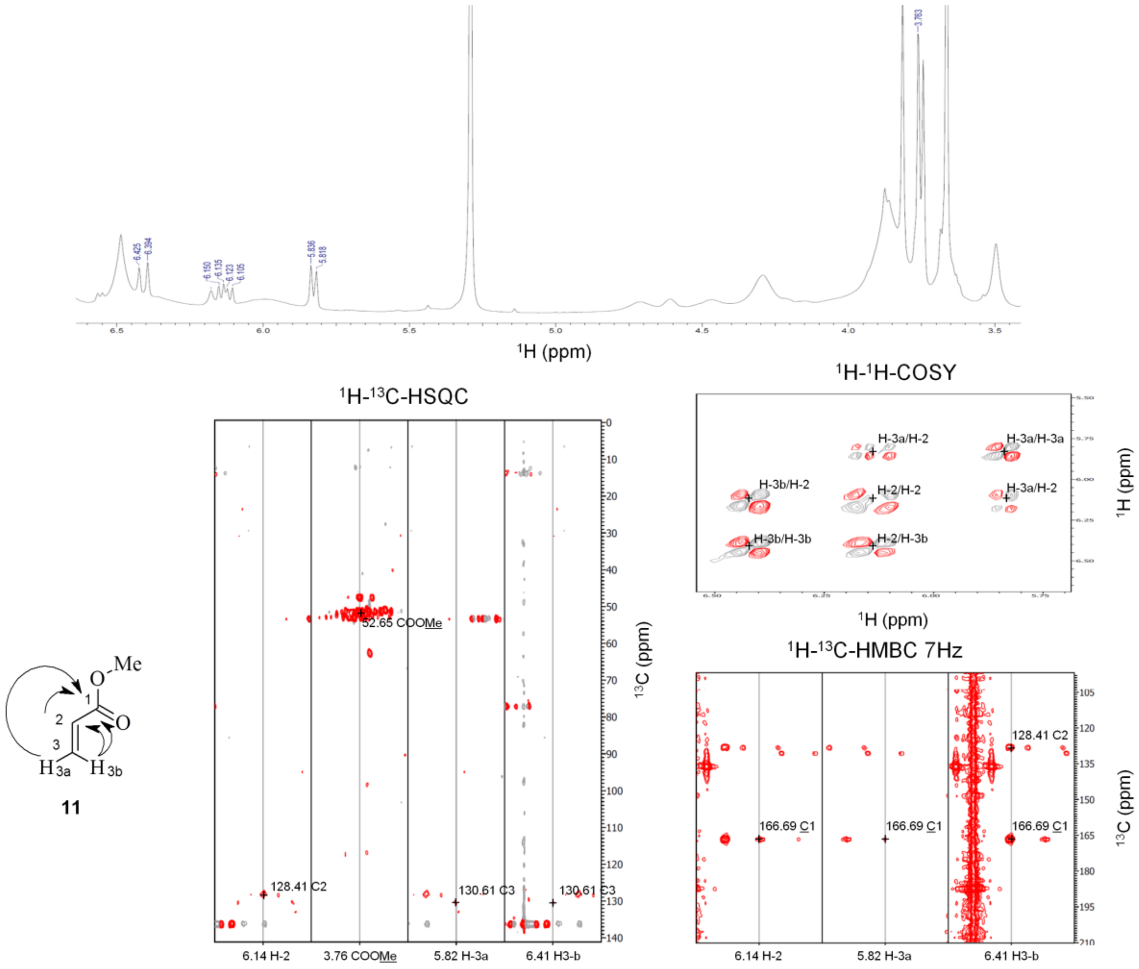

Figure S5. NMR spectra used for the proton and carbon assignments of the compound **11**. For clarity, bi-dimensional HSQC and HMBC spectra are showed as “strip plot” (see legend Figure S4). Key HMBC and COSY cross-peaks detected are indicated on the structure.

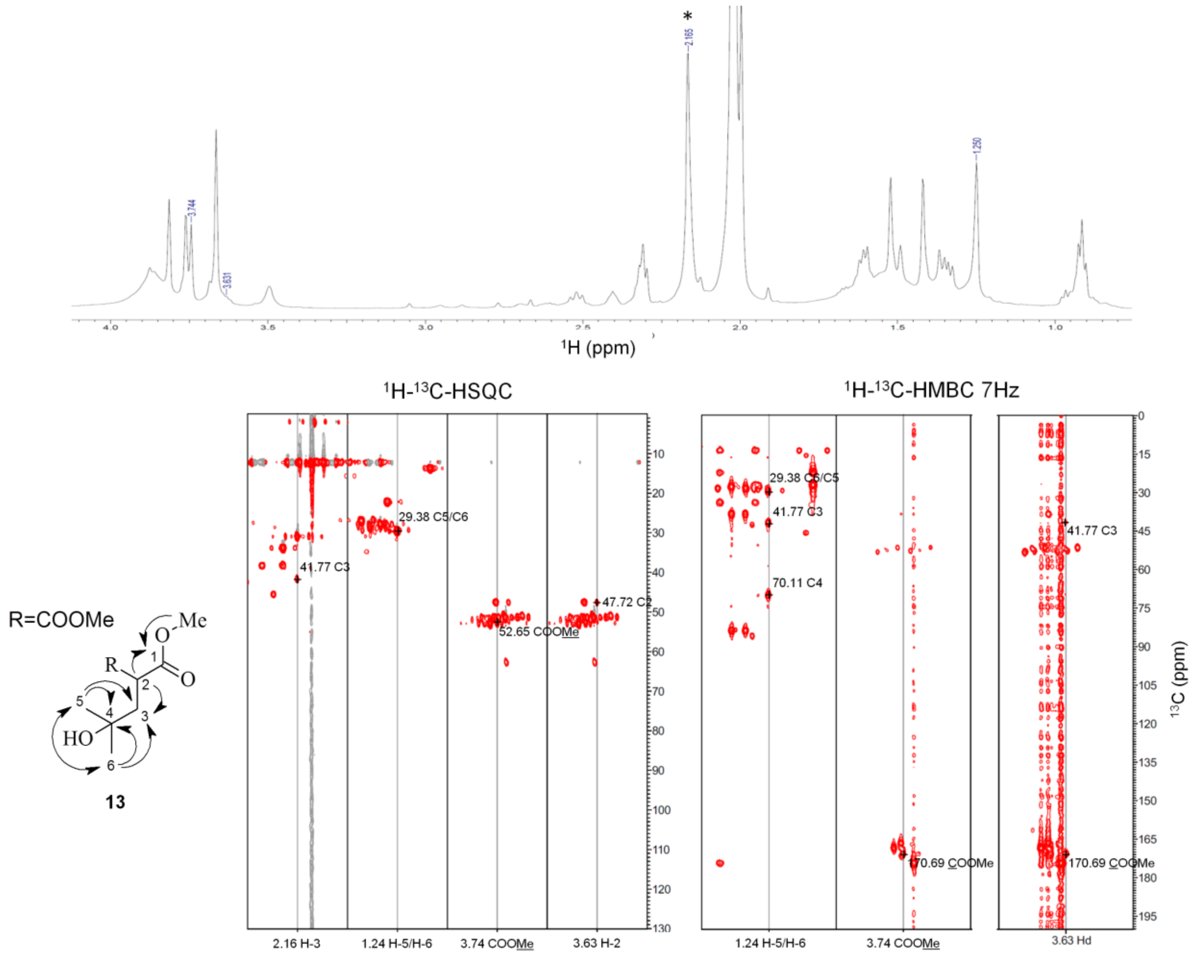

Figure S6. NMR spectra used for the proton and carbon assignments of the compound **13**. For clarity, bi-dimensional HSQC and HMBC spectra are showed as “strip plot” (see legend Figure S4). Key HMBC cross-peaks detected are indicated on the structure. Overlapped signals are marked by an asterisk.

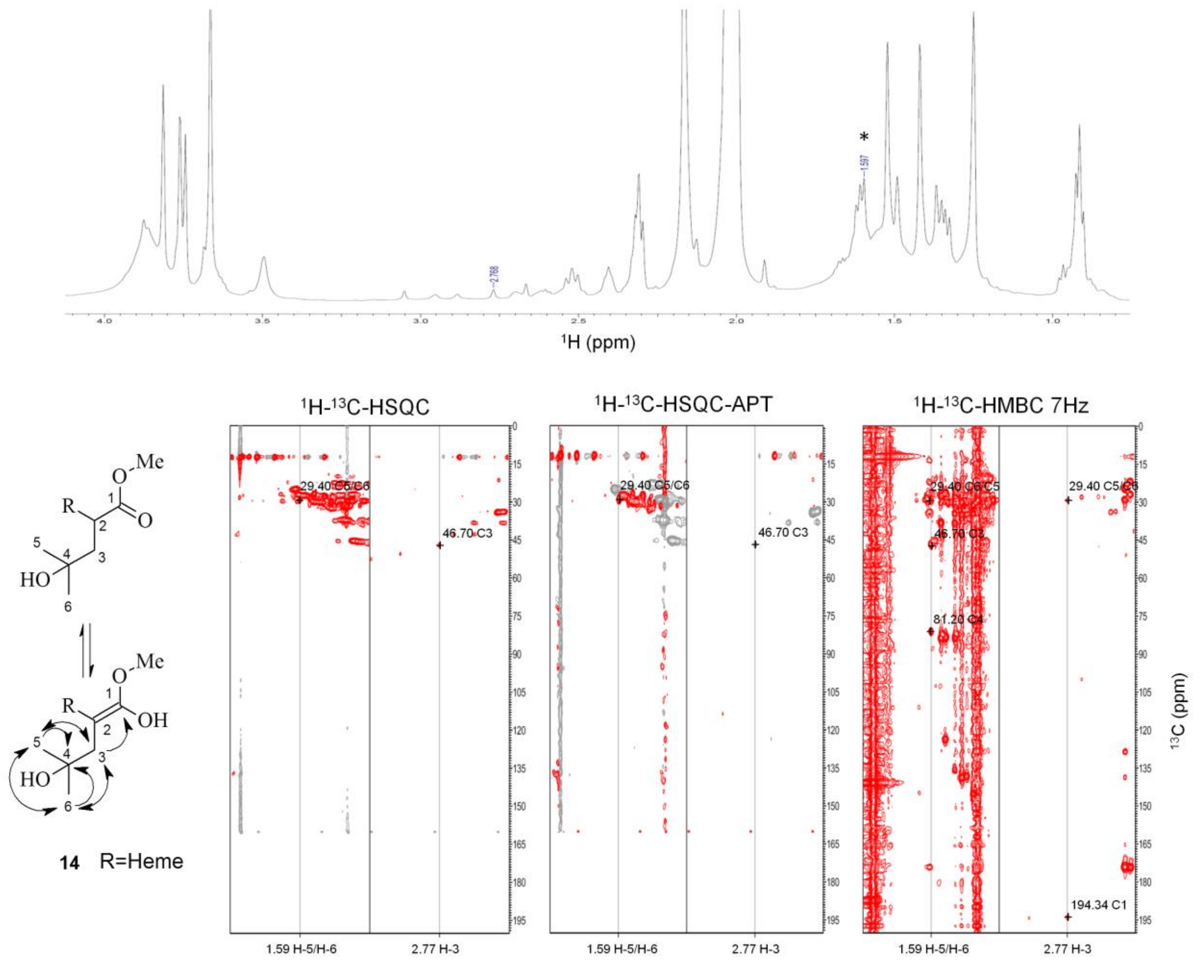

Figure S7. NMR spectra used for the proton and carbon assignments of the compound **14**. For clarity, bi-dimensional HSQC and HMBC spectra are showed as "strip plot" (see legend Figure S4). In the APT-type HSQC, by convention, CH and CH_3 peaks are phased up (red) while CH_2 peaks are phased down (grey). The keto−enol tautomerism of **14** is indicated. Key HMBC and COSY cross-peaks are detected and indicated for the enolic form. Overlapped signals are marked by an asterisk.

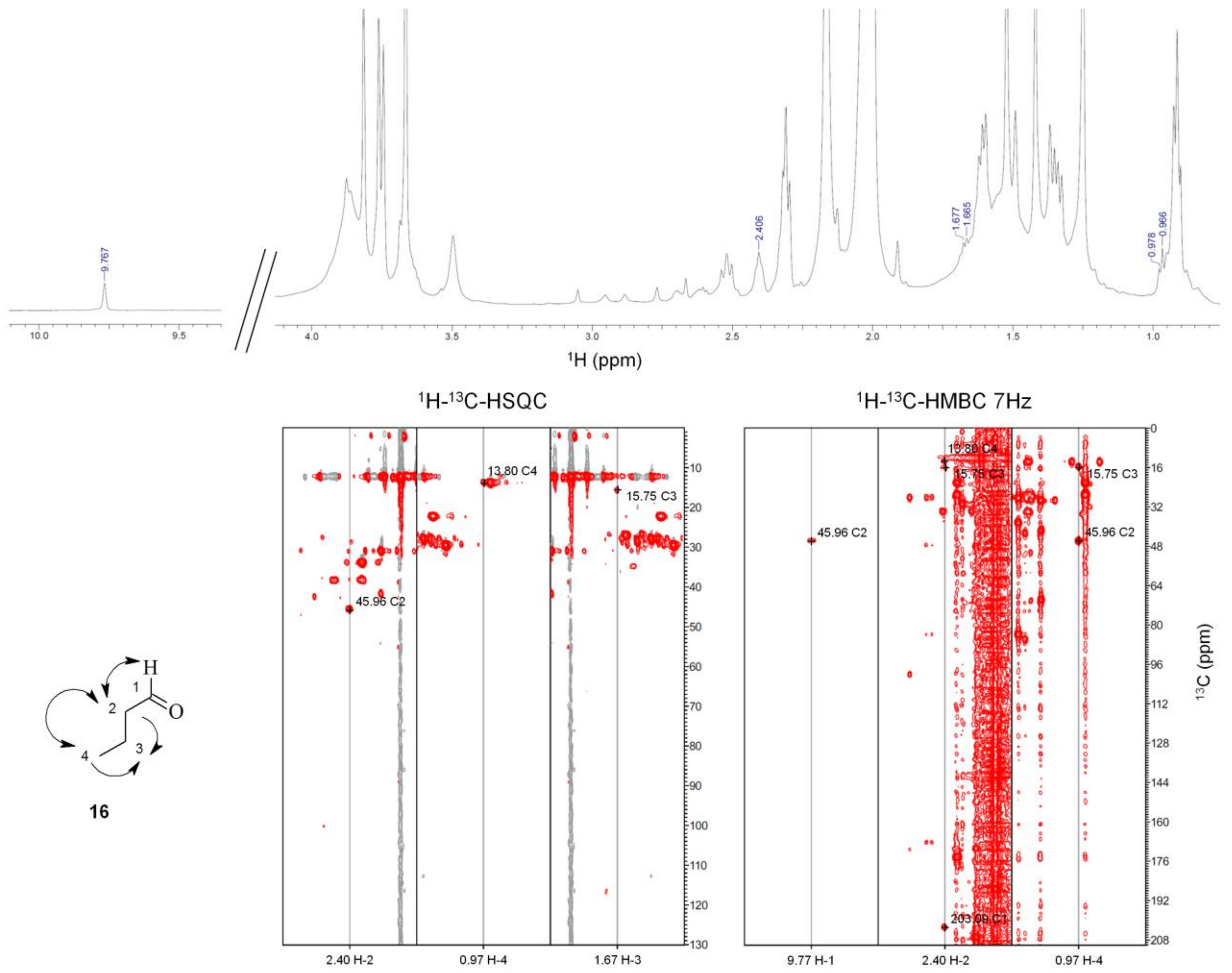

Figure S8. NMR spectra used for the proton and carbon assignments of the compound **16**. For clarity, bi-dimensional HSQC and HMBC spectra are showed as "strip plot" (see legend Figure S4). Key HMBC cross-peaks detected are indicated on the structure.

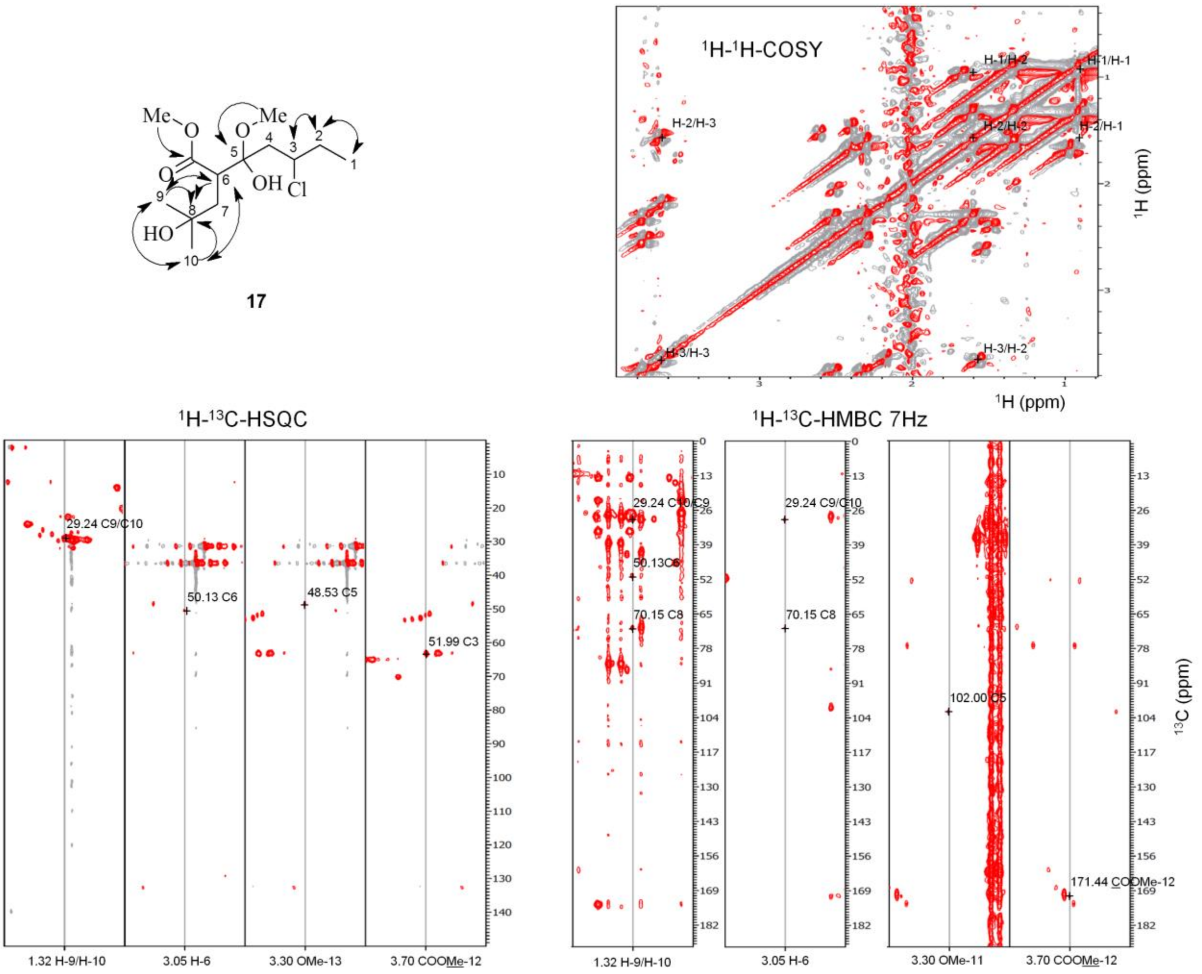

Figure S9. NMR spectra used for the proton and carbon assignments of the compound **17**. For clarity, bi-dimensional HSQC and HMBC spectra are showed as “strip plot” (see legend Figure S4). Key HMBC and COSY cross-peaks detected are indicated on the structure.

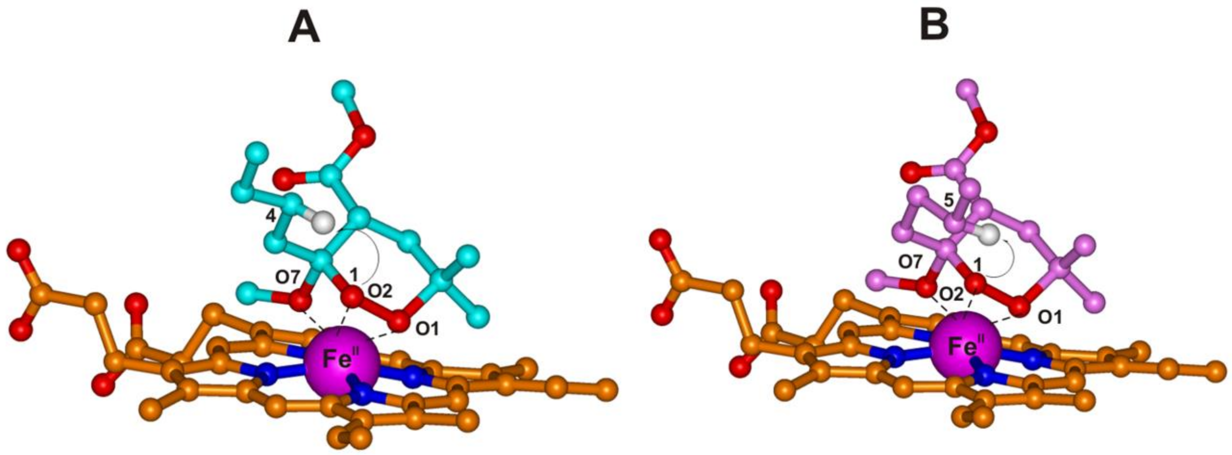

Figure S10. 4a-Fe^{II}-heme (orange) complexes used as starting structures for docking studies. A) **4a** conformer presenting intra-molecular distance suitable for the 1,4-H shift (cyan) and B) **4a** conformer presenting intra-molecular distance suitable for the 1,5-H shift (pink). The molecules are coloured by atom type (O = red; N = blue; Fe = magenta and H = white). The Fe^{II} coordination atoms are labelled. Hydrogens are omitted for sake of clarity, with the exception of those involved as possible partners in the H-shift. Atom numbering refers to the distance between the H-shift possible partners. Iron atom vdW volume is scaled by 50% for clarity of presentation.

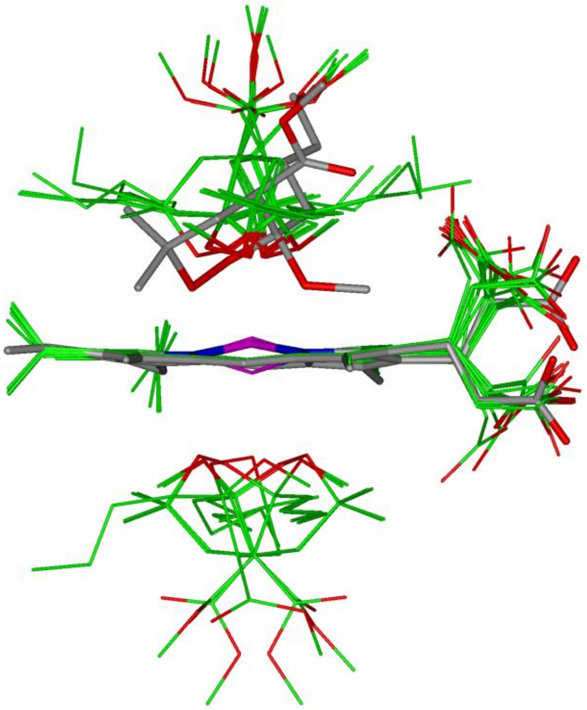

Figure S11. 4a-Fe^{II}-heme complexes resulting from the Monte Carlo/SA docking procedure. The starting complex is coloured in grey; the calculated complexes are coloured in green. Heteroatoms are coloured by atom type (O = red, N = blue, and Fe = magenta); hydrogens are omitted for sake of clarity.

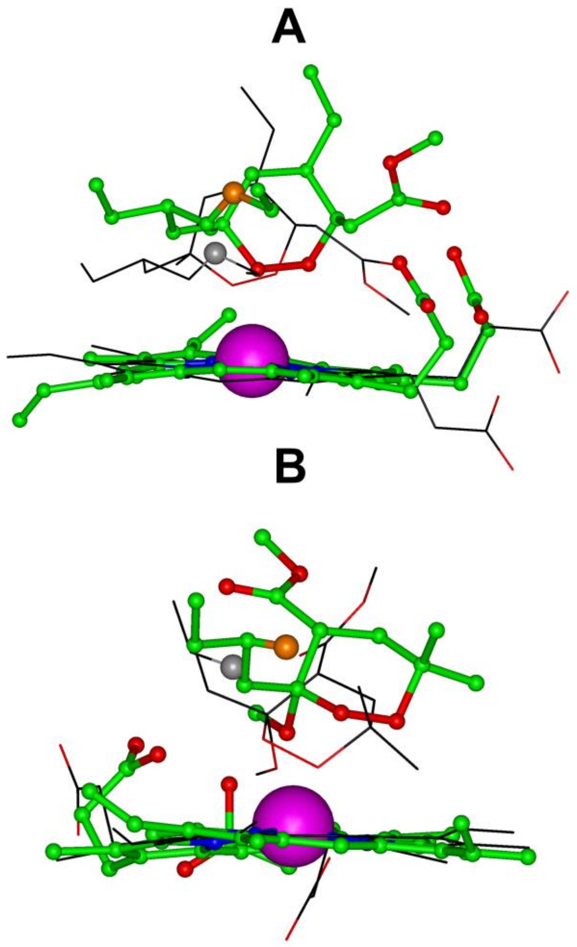

Figure S12. Superimposition between the starting (black) and the final DFT complex (green) of **2** (A) and **4a** (B). Heteroatoms are coloured by atom type (O = red; N = blue; Fe = magenta and H = white). Hydrogens are omitted for sake of clarity with the exception of those involved as possible partner in the radical shift. Iron atom vdW volume is shown scaled by 50% for clarity of presentation. C10 of **2** (A) and the hydrogen of C10 of **4a** (B) are colored in gray in starting complex and in orange in final complex.

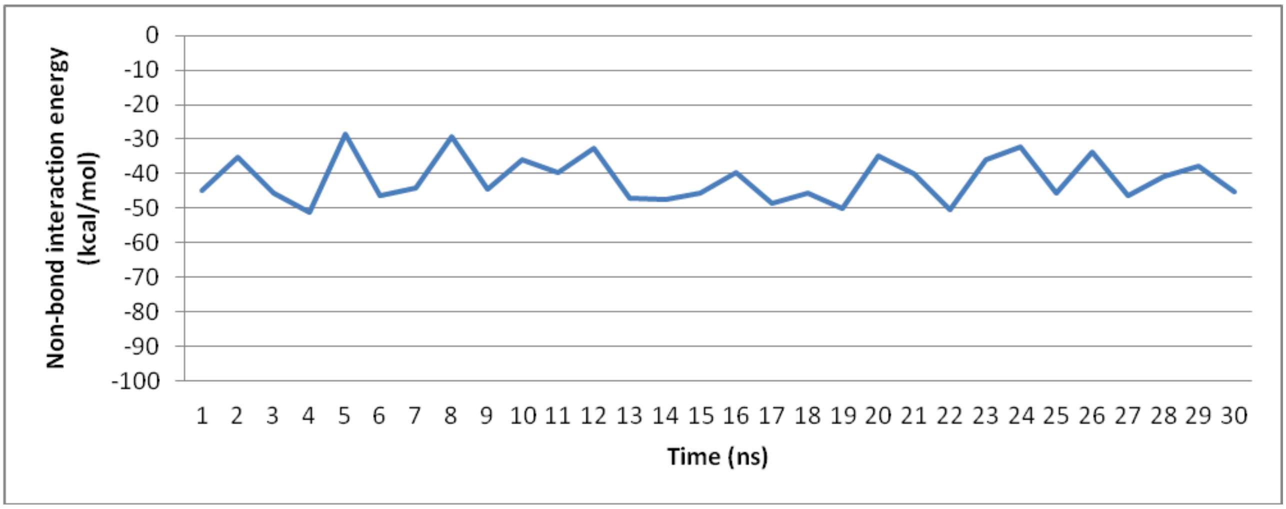

Figure S13. Non-bond interaction energy of **4a**-heme-Fe^{II} complexes obtained every 1 ns during the production phase of MD simulation plotted against the time (ns).

Table S1. 4a/Fe^{II}-heme complexes obtained by dynamic docking studies.

Complex	ΔE_{GM}[a] (kcal/mol)	Putative radical shift
1	0	1,4
2	0.62	1,4
3	1.86	1,4
4	2.67	1,4
5	3.14	1,4
6	3.25	1,4
7	4.33	1,4
8	5.08	1,4
9	6.07	1,4
10	7.24	1,4
11	7.53	-
12	8.61	1,4
13	8.80	1,4
14	9.35	1,4
15	10.20	1,4
16	11.09	1,4
17	11.38	1,4
18	11.63	1,5
19	12.59	1,5

[a]Energy difference from the global minimum (GM) complex

Table S2. Structural features and non-bond interaction energy of **4a**/Fe^{II}-heme complexes produced by 30 ns of molecular dynamics at 298 K.

Time (ns)	O1-Fe^{II} (Å)	O2-Fe^{II} (Å)	O7-Fe^{II} (Å)	H-shift	Non-bond interaction energy (kcal/mol)
1	2.83	2.65	3.87	1,4	-44.9115
2	3.24	3.01	3.99[a]	1,4	-35.1511
3	3.03	2.88	3.65	1,4	-45.7022
4	2.84	2.83	2.77	1,4	-51.0445
5	3.53	3.14	4.04[a]	1,4	-28.5240
6	2.98	2.84	3.18	1,4	-46.1991
7	3.18	3.07	3.49	1,4	-43.9275
8	3.44	3.16	4.16[a]	1,4	-29.3728
9	3.16	2.77	3.70	1,4	-44.4088
10	3.24	2.81	4.24[a]	1,4	-35.9737
11	3.21	3.26	3.43	1,4	-39.7034
12	2.98	3.04	4.34[a]	1,4	-32.7441
13	3.04	2.66	3.27	1,4	-47.1240
14	2.99	2.88	3.66	1,4	-47.3351
15	2.75	3.03	3.54	1,4	-45.5684
16	3.53	3.00	3.63	1,4	-39.6862
17	2.87	3.05	3.37	1,4	-48.4343
18	2.96	2.82	3.56	1,4	-45.5755
19	2.83	2.79	3.47	1,4	-50.2073
20	3.18	2.97	4.43	1,4	-34.9781
21	2.85	2.87	4.02[a]	1,4	-39.9539
22	2.77	2.99	3.41	1,4	-50.2573
23	3.43	2.95	3.79[a]	1,4	-35.7807
24	3.35	2.92	4.13[a]	1,4	-32.2082
25	2.93	2.77	4.05	1,4	-45.5621
26	3.00	3.11	4.28[a]	1,4	-33.7874
27	2.89	2.72	3.94	1,4	-46.2802
28	2.91	2.73	3.82[a]	1,4	-40.7660
29	3.13	2.77	4.35[a]	1,4	-37.8163
30	2.84	2.61	3.33	1,4	-45.1287

[a] Not involved in Fe^{II} coordination.
